# Studies on the Emission of Volatile Organic Compounds from Selected Forest Mushrooms of the Genus *Lactarius* Using Proton-Transfer Reaction Mass Spectrometry

**DOI:** 10.3390/molecules30143000

**Published:** 2025-07-17

**Authors:** Tomasz Wróblewski, Anna Kamińska, Agnieszka Włodarkiewicz

**Affiliations:** Institute of Exact and Technical Sciences, Pomeranian University in Słupsk, ul. Arciszewskiego 22A, 76200 Słupsk, Poland; tomasz.wroblewski@upsl.edu.pl (T.W.); anna.kaminska@upsl.edu.pl (A.K.)

**Keywords:** volatile organic compounds (VOCs), proton-transfer reaction mass spectrometry (PTR-MS), lactarius mushrooms

## Abstract

Forest mushrooms, due to their taste and smell, have been a component of people’s diets since the beginning of time. Unfortunately, there are many inedible or poisonous species of mushrooms that are similar to those that are eaten. For example, the highly valued Boletus edulis is similar to the inedible bitter bolete and the poisonous bolete. In the case of mushrooms of the genus *Lactarius*, such similarities are demonstrated by the delicious tasting *L. deliciosus*, the inedible downy *L. pubescens* and the poisonous cottony *L. torminosus*. This study presents an attempt to classify these three species based on studies of the emission of volatile organic compounds from the volatile headspace using proton-transfer reaction mass spectrometry (PTR-MS). The conducted statistical tests, principal component analysis (PCA) and discriminant analysis revealed significant differences in the concentration of 20 selected protonated VOC molecules for the tested mushroom species. The clear advantages of the PTR-MS technique are that there is no need for special sample preparation and it has rapid measurement capability and high analytical sensitivity. This allows for a quick comparative analysis of VOCs, for example, from different species of forest mushrooms.

## 1. Introduction

Forest mushrooms are valued in many countries for their taste. It is estimated that there are approximately 2300 species of wild edible and medicinal mushrooms in the world [1,2], and in European countries, there are approximately 268 species of forest mushrooms of potential commercial importance [3]. Trade in certain species, such as chanterelles, morels, boletes and delicious milk caps, is an important element of the economy of local rural communities [4,5]. Mushrooms, in addition to their taste, can be a source of valuable compounds such as non-starch polysaccharides, beta-glucans, dietary fiber, protein, ergosterol, statins, minerals and phenolic acids [6,7,8,9]. Unfortunately, some mushrooms also contain toxic substances that can even cause fatal poisoning [10]. The most infamous one is the death cap fly agaric (*Amanita phalloides*), which may be responsible for up to 90% of all fatal mushroom poisonings in the world [11]. The main toxic substances are considered to be amatoxins and phallotoxins, especially α- and β-amanitin [12,13]. These are non-volatile organic compounds (cyclic peptides) composed of several amino acids. In some less poisonous mushrooms, volatile organic compounds (VOCs) may be the main toxins. An example is *Gyromitra esculenta*, which contains the poisonous gyromithrin. Although this mushroom was once quite often eaten, it is now considered to be potentially fatal [14].

The taste of mushrooms results from a huge variety of volatile and non-volatile compounds. In terms of non-volatile components, mushroom flavor is generated by free amino acids and nucleotides, as well as various other compounds such as organic acids, soluble sugars, and polyols [15,16]. However, in the case of mushrooms, their aroma is equally important for the consumer, which is caused by volatile organic compounds (VOCs). Studies on VOC emission profiles of living organisms concern plants, bacteria and fungi. Microfungi [17,18] and, less frequently, macrofungi (mushrooms) are often the object of research. For example, studies of VOC concentrations from seven species of mushrooms [19] showed that the aroma of these species comes mainly from volatile substances with eight carbon atoms, such as 1-octen-3-ol and 1-octen-3-one. Subsequent research has shown that 1-octen-3-one is the main compound causing the mushroom-like odor, while the recognizable odors of various species are caused by other compounds such as fatty acid degradation products, 3-(methylthio) propanal, terpenoids and heterocyclic N-compounds [20,21,22]. Measurements using gas chromatography and olfactometry [23] showed that fatty acid degradation products contributed to the odor of wild edible mushrooms.

Various measurement techniques are used to study the content of chemical compounds in mushrooms, such as gas chromatography (GC), liquid chromatography (LC), infrared and UV-vis spectroscopy, Raman spectrometry and nuclear magnetic resonance (NMR) or mass spectrometry (MS) [24]. MS is most often used in combination with chromatographic methods [25,26]. However, chromatographic methods, in particular gas chromatography, require appropriate preparation of the sample, e.g., concentration in an adsorber, and then its evaporation at an appropriate temperature. This may affect the chemical composition of the tested sample due to the instability of some chemical compounds. An alternative to chromatographic tests may be proton-transfer reaction mass spectrometry (PTR-MS). This technique uses so-called soft chemical ionization, which involves the transfer of a proton from the hydronium ion to the tested molecule. This allows the molecular fragmentation processes that occur in “traditional” mass spectrometers to be limited, e.g., with electron beam ionization. Other advantages of PTR-MS include the fact that there is no need for special sample preparation, its rapid measurement capability and its high analytical sensitivity (of the ppt order) [27,28]. One of the main disadvantages of this VOC testing method is the inability to distinguish and identify compounds with identical molecular weight. Despite this, this method seems to be ideal for comparative research or for determining the so-called volatile fingerprints of the tested samples [29,30].

This paper presents the results of comparative studies of VOC emission profiles of forest cap-type mushrooms using proton-transfer reaction mass spectrometry (PTR-MS). The aim of the research is to check whether there are differences in the mass spectra of VOCs from similar fungi, some of which are edible and others inedible or poisonous. For this purpose, the following species of fungi from the *Lactaria* were selected: *L. pubescens*, *L. deliciosus* and *L. torminosus*.

## 2. Results and Discussions

**Figure 1:**
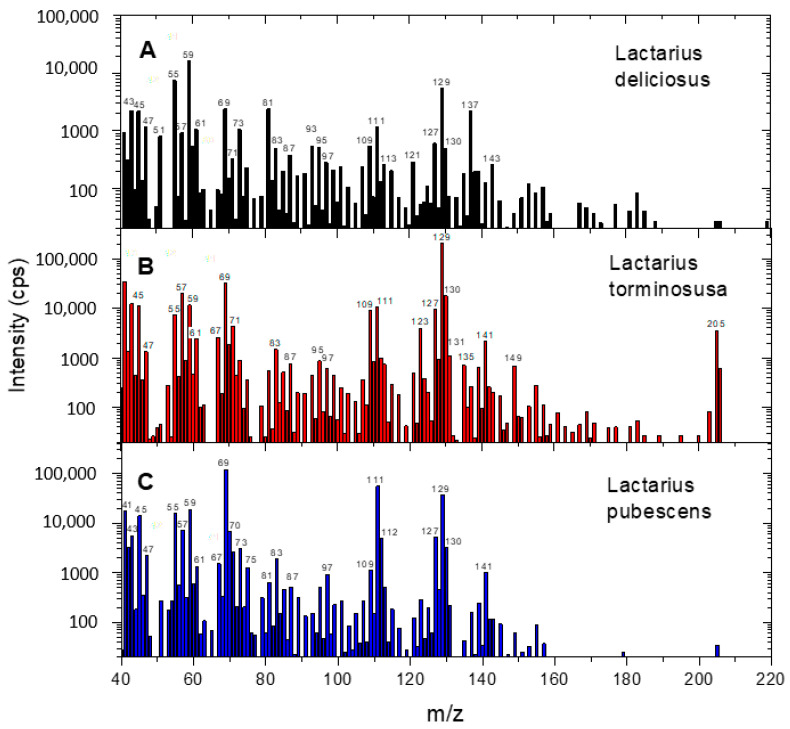
Comparison of VOC mass spectra for *Lactarius deliciosus* (**A**), *Lactarius torminosus* (**B**) and *Lactarius pubescens* (**C**).

Examples of comparative VOC mass spectra for *L. deliciosus*, *L. torminosus* and *L. pubescens* are shown in Figure 1. Please note that the mass spectra are shifted by 1 amu due to protonation of the molecules in the drift chamber. Overall, it can be concluded that the mass spectra are quite similar. The registered ions correspond to various alcohols, aldehydes, hydrocarbons, acids, esters and terpenoids [31]. However, in the mass spectra, a quite strong signal was recorded for *m*/*z* 205 only in the case of *L. torminosus* (C), which corresponds to a protonated sesquiterpene with the formula C_15_H_24_. This effect was reproducible for all samples.

To determine whether there are significant differences in the VOC emission profiles of the tested mushroom species, 20 *m*/*z* values were selected for further research: 59, 61, 63, 69, 71, 73, 81, 83, 95, 101, 109, 111, 121, 123, 127, 129, 137, 155 and 205. When selecting the *m*/*z* value, we were guided by the high ionic signal or visible differences in this signal between the tested fungal species, as in the case of *m*/*z* = 205.

All tests (Wilks’ Lambda, Pillai’s Trace, Hotelling, Roy’s) indicate significant differences between groups (all *p*-values are very low, indicating statistical significance). The *p*-values are less than 0.05 (or selected significance levels), which indicates that there are statistically significant differences between the fungal groups with respect to the intensity of the analyzed masses. Post hoc tests were performed to find out which specific variables (*m*/*z*) contribute to these differences.

The results of these analyses indicate that the intensities of many of the *m*/*z* values differ significantly between the tested fungal species (Table 1). *L. torminosus* (group C) shows the greatest differences compared to *L. pubescens* (B) (group A) and *L. deliciosus* (group B) for most of the analyzed *m*/*z* values. The lack of significant differences between group A and group B for some masses suggests that these two species are more similar to each other in terms of the intensity of selected chemical compounds.

**Figure 2:**
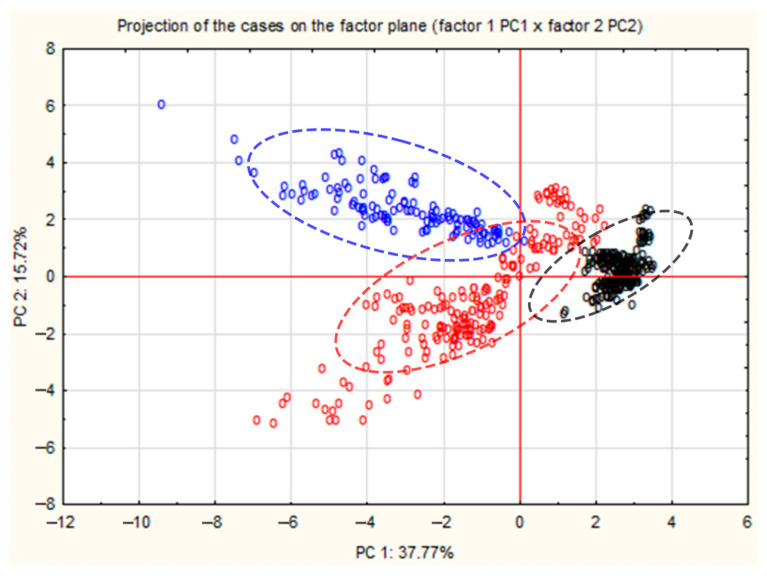
PCA graph for protonated VOCs taken from *Lactarius pubescens* (blue), *Lactarius deliciosus* (red) and *Lactarius torminosus* (black). Ellipses indicate approximate 90% confidence intervals.

The results of the PCA are presented in Figure 2. Based on the plot of the projection of cases onto the plane of the first and second principal components (PC1 and PC2) for three species of fungi, the following conclusions can be drawn:

The first principal component (PC1) explains 37.77% of the total variance in the data.

The second principal component (PC2) explains 15.72% of the total variance in the data.

Together, PC1 and PC2 explain 53.49% of the total variance in the data, which means that more than half of the information contained in the original variables (*m*/*z*) was captured by these two principal components.

The fungal samples are distributed across the PC1 and PC2 planes in a manner that suggests some species-specific grouping. *L. torminosus* (group C) appears to be clearly separated from *L. pubescens* (group A) and *L. deliciosus* (group B) along the PC1 component. *L. pubescens* and *L. deliciosus* have some overlap, but also show some distinct clusters along the PC2 component. The PC1 component probably captures the greatest differences between *L. torminosus* and the other two fungal species. The PC2 component additionally differentiates the *L. pubescens* from the *L. deliciosus*, although it differentiates the *L. torminosus* to a lesser extent than PC1. The clear separation of *L. torminosus* from other species at the PC1 and PC2 plane suggests that this species has unique *m*/*z* mass profiles that are well captured by these principal components. Some overlap between *L. pubescens* and *L. deliciosus* indicates that these species have more similar *m*/*z* profiles but can still be differentiated using PC2. These results can be used to further identify specific biomarkers for particular fungal species and have potential applications in environmental and industrial research.

The discriminant analysis conducted for three species of fungi, *L. pubescens* (group A), *L. deliciosus* (group B) and *L. torminosus* (group C), showed that the model consisting of 20 variables is highly effective in the classification of these species (Table 2). Wilks’ Lambda is 0.00977, indicating a very strong rejection of the null hypothesis of group equality, with an F-value of 233.92 and *p* < 0.0000. The classification matrix (Table 3) (also known as a confusion matrix) that presents the relationship between the actual (observed) and predicted classifications produced by a statistical model illustrates how accurately the discriminant analysis model—based on PTR-MS data for 20 selected *m*/*z* values—was able to assign mushroom samples to their correct species (*L. pubescens*—A; *L. deliciosus*—B; and *L. torminosus*—C).

Each row of the matrix corresponds to the true group, while each column represents the predicted classification by the model. The diagonal cells contain the number of correctly classified samples, whereas off-diagonal cells would indicate misclassifications.

In our results, the model demonstrated excellent performance, 98.33% correct classification for group A, 100% for group B and 100% for group C, leading to an overall classification accuracy of 99.63%. This confirms the model’s high discriminative power based on the selected VOC emission profiles.

Based on the results of the summary of the discriminant function analysis, we can assess the importance of individual variables. The summary results indicate that most of the variables have very low *p*-values (<0.05), which means that they are statistically significant and played a significant role in the classification. However, the variable *m*/*z* 61 has a *p*-value of 0.428244 and *m*/*z* 121 has a *p*-value of 0.711548. These *p*-values are significantly higher than 0.05, which means that these variables did not play a significant role in the discriminant analysis. Additionally, the analysis of tolerance coefficients (1-Toler. (R-quad)) indicates variables that have a low tolerance value, which may suggest a problem with collinearity, but in the context of their role in the model, *p*-values are crucial. On this basis, it can be conclude that *m*/*z* 61 and *m*/*z* 121 did not have a significant impact on distinguishing groups in the model.

**Figure 3:**
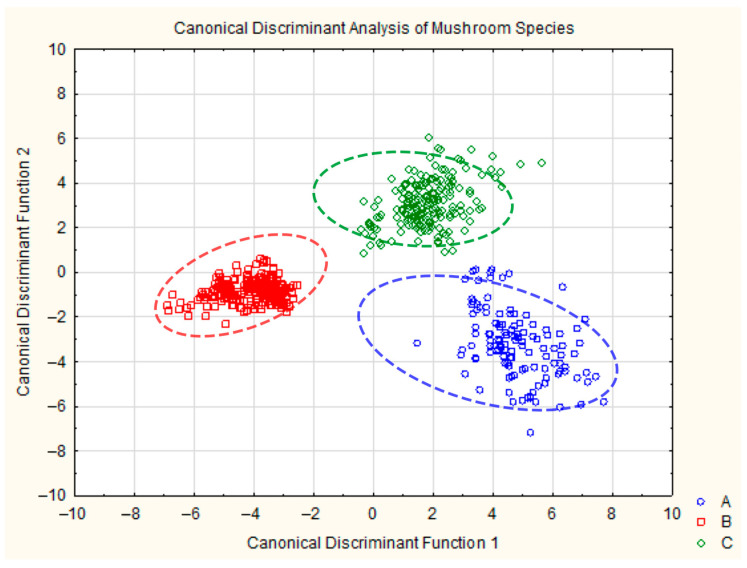
Plot of canonical discrimination analysis for *Lactarius pubescens* (blue), *Lactarius deliciosus* (red), and *Lactarius torminosus* (green). Ellipses indicate approximate 90% confidence intervals.

The scatterplot of the canonical values shows distinct clusters for each of the three fungal groups, confirming the classification results. Each group is well separated in the canonical space, demonstrating the effectiveness of the discriminant model in distinguishing these species (Figure 3).

## 3. Materials and Methods

### 3.1. Mushroom Samples

**Figure 4:**
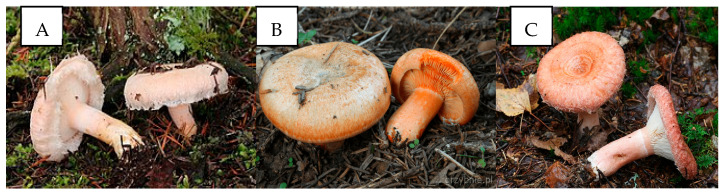
Appearance of *Lactarius pubescens* (**A**), *Lactarius deliciosus* (**B**) and *Lactarius torminosus* (**C**).

Three species of fungi from the genus *Lactarius* (*Russulaceae Lotsy*) were selected for the study—*L. pubescens*, *L. deliciosus* and *L. torminosus* (Figure 4). Due to its burning taste, mossy *L. pubescens* is considered an inedible mushroom [32]. *L. torminosus* is considered to be the most similar to it. Despite the fact that in some countries, e.g., Russia, it is eaten after thermal treatment, in most countries, *L. torminosus* is considered a poisonous mushroom [32]. On the contrary to *L. torminosus*, *L. deliciosus* is an edible mushroom and highly valued due to its delicious taste. A characteristic feature of *Lactarius* mushrooms is the latex (“milk”), which is exuded only after damage to the flesh, especially in the gills. *L. pubescens* and *L. torminosus* produce white latex, while *L. deliciosus* exudes an orange latex. The cap of *L. deliciosus* is smooth, whereas the caps of *L. pubescens* and *L. torminosus* are covered with a felt-like skin. All mushrooms came from forest areas located in northern Poland on the border of the Pomeranian Voivodeship and the West Pomeranian Voivodeship, near two villages—Łętowo and Korzybie. The mushrooms were collected in late summer and autumn in 2020–2023.

### 3.2. VOC Measurements

In this work, a high-sensitivity proton-transfer reaction mass spectrometer from Ionicon Analytik from Austria was employed for comparative analysis of the VOC emission profiles of three species of fungi of the Lactaria genus. The measurement methodology was similar to that presented in [33]. Briefly, mushroom samples weighing 50 g were placed in a 250 mL glass vessel and covered with aluminum foil. It should be emphasized that the mushrooms were delivered to the laboratory on the same day they were collected. A tube was inserted into the vessel through a small hole in the foil, through which air from above the samples was sucked into the drift chamber of the PTR-MS spectrometer, where proton-transfer reactions between hydronium ions and VOC molecules from the samples take place. The research used 30 samples of *L. pubescens*, 50 samples of *L. deliciosus* and 45 samples of *L. torminosus*, and four scans were performed for each sample in the range from *m*/*z* 21 to 300. Measurements were made at a pressure in the spectrometer’s drift chamber of 2.2 bar, voltage of 600 V and temperature of 60 °C. To limit the VOC condensation processes in the tube through which the samples were fed to the spectrometer, it was heated to a temperature of 60 °C. Before each actual measurement, background spectra were also recorded to control the possible memory effect. In all cases, the background was practically the same and typical signals originating primarily from the ion source were recorded in its spectrum. VOC concentrations calculated in the PTR-MS Viewer 3.2.12 program were used for further analysis. Since the exact chemical composition of the atmosphere above the mushrooms was not known, and therefore it was not possible to assign a value for the reaction rate coefficient *k* between the hydronium ion and specific VOC molecules needed to accurately determine the concentration, the *k* value was selected as 2.0·10^−9^ cm^3^ s^−1^ in the PTR-MS Viewer program; this was the same for all *m*/*z* values. It is true that it is estimated that adopting such an equal value of *k* may cause an error in determining a concentration of about 30%, but in comparative studies, it does not matter. It is important that all measurements were made under the same conditions [34,35].

### 3.3. Statistical Methods

The statistical analysis was performed in the Statistica 13 program (Statistica 2017, Cary, NC, USA) using the method from [36]. The statistical analysis included basic statistical parameters and multidimensional cluster analysis (Ward’s method, Euclidean distance). To determine the type of distribution of variables, the Kolmogorov–Smirnov test was used. If the sample had a normal distribution, ANOVA analysis with the RIR Tukey test was used. In cases with a lack of normal distribution, the non-parametric equivalent was used: the Kruskal–Wallis test and then the Dunn post hoc test.

In order to detect possible differences in VOC emission profiles of the tested mushroom species, Principal Component Analysis (PCA) and canonical discriminant analysis were also used.

## 4. Summary and Conclusions

This study presents the results of measurements of volatile organic compounds emitted from three species of fungi of the *Lactarius* genus—*L. pubescens*, *L. deliciosus* and *L. torminosus*—using proton-transfer reaction mass spectrometry. A simple analysis of differences in ion signal intensity for selected mass-to-charge ratios (*m*/*z*) in the “raw” mass spectra of VOCs from the tested mushrooms does not allow for unambiguous identification of species. However, it can be noticed that in the case of *L. torminosus*, a clear signal appeared in the mass spectrum at an *m*/*z* value equal to 205. This most likely corresponds to a protonated molecule from the sesquiterpene group with the molecular formula C_15_H_24_. For the other two species, the signal intensity for this *m*/*z* value was clearly lower. It turned out to be impossible to distinguish between *L. deliciosus* and *L. pubescens* on this basis. Therefore, statistical analyses were conducted, which showed that, in most cases, there are significant differences in the concentration of VOCs in the volatile headspace of mushrooms for 20 selected mass-to-charge *m*/*z* ratios. *L. torminosus* shows the greatest differences compared to *L. pubescens* and *L. deliciosus* for most of the analyzed *m*/*z* values. The lack of significant differences between group A and group B for some masses suggests that these two species are more similar to each other in terms of the intensity of selected chemical compounds.

As part of the PCA, it was also found that the distribution of results for individual mushroom species on the PC1 and PC2 planes suggests some grouping depending on the species. *L. torminosus* appears to be clearly separated from *L. pubescens* and *L. deliciosus* along the PC1 component. *L. pubescens* and *L. deliciosus* have some overlap, but also show some distinct clusters along the PC2 component.

Discriminant analysis turned out to be equally helpful in distinguishing the tested fungal species based on PTR-MS measurements. On the scatterplot of canonical values, distinct clusters can be seen for each of the three groups of fungi, which enables their classification.

Based on the results obtained, it can be concluded that the use of the PTR-MS technique to distinguish similar mushroom species based on differences in VOC concentrations in the volatile headspace above the samples is highly effective, in particular using various statistical analyses. However, it should be remembered that such analyses do not guarantee 100% identification of a given mushroom species due to their statistical nature.

## Figures and Tables

**Table 1:** Results of post hoc tests (Tukey’s HSD). A—*Lactarius pubescens*; B—*Lactarius deliciosus*; C—*Lactarius torminosus*.

*m*/*z*	Important Differences
57	A-C, B-C
59	A-C, B-C
61	A-C, B-C
63	A-C, B-C, A-B
69	A-C, B-C, A-B
71	A-C, B-C
73	A-C, B-C
81	A-C, B-C
83	A-C, B-C
95	A-C, B-C, A-B
101	A-C, B-C, A-B
109	A-C, B-C, A-B
111	A-C, B-C, A-B
121	A-C, B-C, A-B
123	A-C, B-C, A-B
127	A-C, B-C, A-B
129	A-C, B-C, A-B
137	A-C, B-C, A-B
155	A-C, B-C, A-B
205	A-C, B-C

**Table 2:** Summary of discriminant function analysis. Variables in the model: 20; grouping: mushroom species (3 groups); Wilks’ Lambda: 00977; adj. F (40.1026) = 233.92; *p* < 0.0000.

N = 535	Wilks’ Lambda	Partial Wilks’ Lambda	F-Value (2.513)	*p*	Tolerance	1-Tolerance (R-Squared)
*m*/*z* 57	0.014	0.715	102.245	0.000	0.229	0.771
*m*/*z* 59	0.013	0.764	79.313	0.000	0.544	0.456
*m*/*z* 61	0.010	0.997	0.850	0.428	0.719	0.281
*m*/*z* 63	0.010	0.937	17.276	0.000	0.520	0.480
*m*/*z* 69	0.011	0.877	35.885	0.000	0.019	0.981
*m*/*z* 71	0.011	0.885	33.346	0.000	0.240	0.760
*m*/*z* 73	0.012	0.847	46.488	0.000	0.283	0.717
*m*/*z* 81	0.012	0.840	48.875	0.000	0.236	0.764
*m*/*z* 83	0.011	0.869	38.632	0.000	0.061	0.939
*m*/*z* 95	0.012	0.845	46.987	0.000	0.389	0.611
*m*/*z* 101	0.010	0.972	7.365	0.001	0.482	0.518
*m*/*z* 109	0.012	0.787	69.225	0.000	0.497	0.503
*m*/*z* 111	0.011	0.929	19.530	0.000	0.021	0.979
*m*/*z* 121	0.010	0.999	0.341	0.712	0.821	0.179
*m*/*z* 123	0.010	0.946	14.518	0.000	0.324	0.676
*m*/*z* 127	0.010	0.997	0.777	0.461	0.160	0.840
*m*/*z* 129	0.010	0.968	8.539	0.000	0.442	0.558
*m*/*z* 137	0.010	0.968	8.393	0.000	0.433	0.567
*m*/*z* 155	0.010	0.994	1.658	0.191	0.466	0.534
*m*/*z* 205	0.010	0.945	14.832	0.000	0.312	0.688

**Table 3:** Classification matrix. Rows: Observed classification. Columns: Predicted classification.

Group	*p*-Value	Correct Percent	A	B	C
A	*p* = 0.22430	98.33	118	0	2
B	*p* = 0.42243	100.00	0	226	0
C	*p* = 0.35327	100.00	0	0	189
total		99.63	118	226	191

## Data Availability

The original contributions presented in this study are included in the article. Further inquiries can be directed to the corresponding author.
